# (*Z*)-*N*-[2-(*N*′-Hy­droxy­carbamimido­yl)phen­yl]acetamide

**DOI:** 10.1107/S1600536813003371

**Published:** 2013-02-09

**Authors:** Hoong-Kun Fun, Chin Wei Ooi, Dinesha, S. Viveka, G.K. Nagaraja

**Affiliations:** a X-ray Crystallography Unit, School of Physics, Universiti Sains Malaysia, 11800 USM, Penang, Malaysia; b Department of Pharmaceutical Chemistry, College of Pharmacy, King Saud University, PO Box 2457, Riyadh 11451, Saudi Arabia; c Department of Chemistry, Mangalore University, Karnataka, India

## Abstract

The asymmetric unit of the title compound, C_9_H_11_N_3_O_2_, contains two mol­ecules (*A* and *B*), which exist in *Z* conformations with respect to their C=N double bond. The dihedral angles between the benzene ring and the pendant hy­droxy­carbamimidoyl and acetamide groups are 28.58 (7) and 1.30 (5)°, respectively, in mol­ecule *A* and 25.04 (7) and 27.85 (9)°, respectively, in mol­ecule *B*. An intra­molecular N—H⋯N hydrogen bond generates an *S*(6) ring in both mol­ecules. Mol­ecule *A* also features an intra­molecular C—H⋯O inter­action, which closes an *S*(6) ring. In the crystal, the mol­ecules are linked by N—H⋯O, N—H⋯N, O—H⋯O, O—H⋯N, C—H⋯O and C—H⋯N hydrogen bonds and C—H⋯π inter­actions, generating a three-dimensional network.

## Related literature

For background and applications of amidoximes, see: Clapp (1976 ▶, 1984 ▶); Jochims (1996 ▶); Fylaktakidou *et al.* (2008 ▶); Mansuy & Boucher (2004 ▶); Kontogiorgis & Hadjipavlou-Litina (2002 ▶); Wang *et al.* (2002 ▶). For hydrogen-bond motifs, see: Bernstein *et al.* (1995 ▶). For bond-length data, see: Allen *et al.* (1987 ▶). For the stability of the temperature controller used for the data collection, see: Cosier & Glazer (1986 ▶).
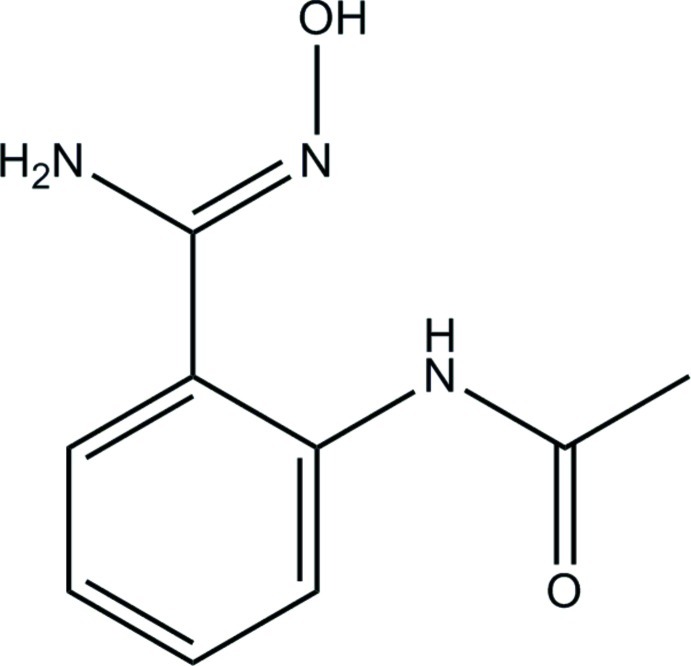


## Experimental

### 

#### Crystal data

C_9_H_11_N_3_O_2_
*M* *_r_* = 193.21
Triclinic, 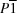

*a* = 8.7813 (12) Å
*b* = 9.5432 (13) Å
*c* = 11.9770 (15) Å
α = 80.722 (2)°
β = 78.531 (2)°
γ = 70.181 (2)°
*V* = 920.6 (2) Å^3^
*Z* = 4
Mo *K*α radiation
μ = 0.10 mm^−1^
*T* = 100 K
0.35 × 0.20 × 0.05 mm

#### Data collection

Bruker APEX DUO CCD diffractometer
Absorption correction: multi-scan (*SADABS*; Bruker, 2009 ▶) *T*_min_ = 0.965, *T*_max_ = 0.995
12849 measured reflections
4680 independent reflections
3815 reflections with *I* > 2σ(*I*)
*R*_int_ = 0.031

#### Refinement

*R*[*F* ^2^ > 2σ(*F* ^2^)] = 0.042
*wR*(*F* ^2^) = 0.116
*S* = 1.07
4680 reflections
287 parameters
H atoms treated by a mixture of independent and constrained refinement
Δρ_max_ = 0.34 e Å^−3^
Δρ_min_ = −0.26 e Å^−3^

### 

Data collection: *APEX2* (Bruker, 2009 ▶); cell refinement: *SAINT* (Bruker, 2009 ▶); data reduction: *SAINT*; program(s) used to solve structure: *SHELXTL* (Sheldrick, 2008 ▶); program(s) used to refine structure: *SHELXTL*; molecular graphics: *SHELXTL*; software used to prepare material for publication: *SHELXTL* and *PLATON* (Spek, 2009 ▶).

## Supplementary Material

Crystal structure: contains datablock(s) global, I. DOI: 10.1107/S1600536813003371/hb7034sup1.cif

Structure factors: contains datablock(s) I. DOI: 10.1107/S1600536813003371/hb7034Isup2.hkl

Supplementary material file. DOI: 10.1107/S1600536813003371/hb7034Isup3.cml

Additional supplementary materials:  crystallographic information; 3D view; checkCIF report

## Figures and Tables

**Table 1:** Hydrogen-bond geometry (Å, °) *Cg*1 and *Cg*2 are the centroids of the C1*A*–C6*A* and C1*B*–C6*B* benzene rings, respectively.

*D*—H⋯*A*	*D*—H	H⋯*A*	*D*⋯*A*	*D*—H⋯*A*
N3*A*—H3*NA*⋯N1*A*	0.883 (19)	2.048 (19)	2.7463 (18)	135.2 (15)
N3*B*—H3*NB*⋯N1*B*	0.86 (2)	1.963 (19)	2.6798 (17)	139.7 (17)
N2*B*—H1*NB*⋯O2*B* ^i^	0.88 (2)	2.10 (2)	2.9725 (17)	173.0 (17)
N2*A*—H2*NA*⋯O2*A* ^ii^	0.87 (3)	2.53 (2)	3.3004 (17)	149.0 (17)
N2*A*—H2*NA*⋯N2*B* ^iii^	0.87 (3)	2.54 (2)	3.2522 (18)	139.6 (17)
N2*B*—H2*NB*⋯O2*A* ^iv^	0.903 (19)	2.12 (2)	2.8900 (17)	142.1 (16)
O1*A*—H1*OA*⋯O1*B*	0.933 (19)	1.844 (19)	2.7733 (15)	173.9 (18)
O1*B*—H1*OB*⋯N1*A* ^v^	0.951 (18)	1.809 (18)	2.7597 (15)	177.0 (17)
C2*A*—H2*AA*⋯O2*A*	0.95	2.22	2.8556 (17)	123
C5*A*—H5*AA*⋯O2*B* ^vi^	0.95	2.55	3.2773 (17)	133
C9*A*—H9*AC*⋯N1*B*	0.98	2.56	3.499 (2)	160
C4*A*—H4*AA*⋯*Cg*2^ii^	0.95	2.95	3.7524 (16)	143
C3*B*—H3*BA*⋯*Cg*1^vii^	0.95	2.88	3.6645 (17)	141

Symmetry codes: (i) 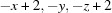; (ii) 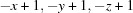; (iii) 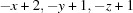; (iv) 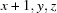; (v) 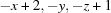; (vi) 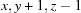; (vii) 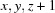.

**Fig. 1.**
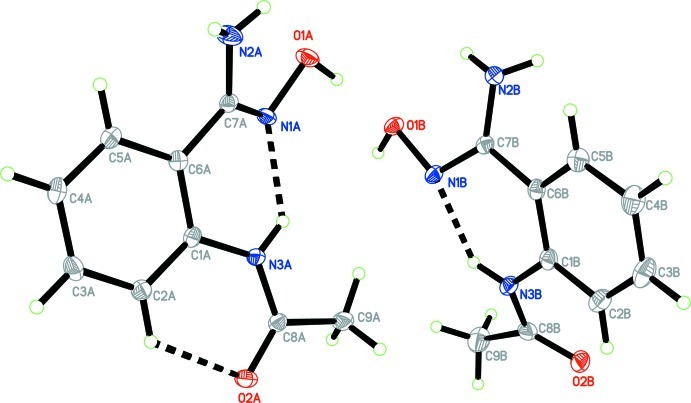
The molecular structure of the title compound, showing 50% probability displacement ellipsoids. Dashed lines indicate the intramolecular hydrogen bonds.

**Fig. 2.**
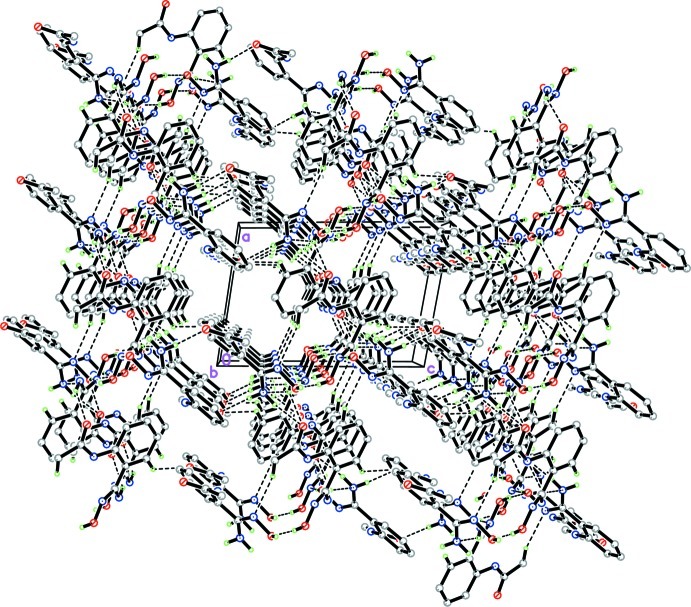
The crystal packing of the title compound, viewed along the b axis. H atoms not involved in the intermolecular interactions (dashed lines) have been omitted for clarity.

## supplementary crystallographic information

### Comment

Amidoximes are bi-functional molecules exhibiting a rich and diverse chemistry and provides the shortest way to reach certain heterocycles, such as 1,2,4-oxadiazoles (Clapp, 1976, 1984; Jochims, 1996). They are also considered interesting molecules in view of their biological applications (Fylaktakidou *et al.*, 2008). Their ability to release NO or nitrites *in vitro* and *in vivo* experiments has recently attracted attention (Mansuy *et al.*, 2004; Kontogiorgis *et al.*, 2002). The discovery that nitric oxide (NO) acts as an important mediator of smooth muscle relaxation has led us to the preparation and testing of a wide variety of compounds with the aim of finding suitable new NO-donors. In the process, the title compound, (*Z*)-*N*-(2-(*N*'-hydroxycarbamimidoyl) phenyl)acetamide (Wang *et al.*, 2002) was prepared.

The title compound consist of two crystallographically independent molecules (A and B) as shown in Fig. 1. The molecules exist in *Z* configuration with respect to the C7A ═N1A and C7B ═N1B double bonds. The intramolecular N3—H3···N1 hydrogen bonds (Table 1) form S(6) ring motifs (Bernstein *et al.*, 1995) in both molecules. Molecule A is stabilized by an additional intramolecular C2A—H2AA···O2A hydrogen bond (Table 1) which also generates an S(6) ring motif (Bernstein *et al.*, 1995). The bond lengths (Allen *et al.*, 1987) and angles are within normal ranges.

In the crystal structure (Fig. 2), the molecules are linked *via* N2B—H1NB···O2B, N2A—H2NA···O2A, N2A—H2NA···N2B, N2B—H2NB···O2A, O1A—H1OA···O1B, O1B—H1OB···N1A, C5A—H5AA···O2B and C9A—H9AC···N1B hydrogen bonds (Table 1) into a three dimensional network The crystal is further consolidated by C4A—H4A···*Cg2* and C3B—H3BA···*Cg1* interactions (Table 1), where *Cg1* and *Cg2* are the centroids of benzene rings (C1A–C6A and C1B–C6B), respectively.

### Experimental

Equimolar amount of *N*-(2-cyanophenyl)acetamide (10 mmol) and NH_2_OH.HCl (10 mmol) were dissolved in a minimum amount of methanol(10 ml)-water (5 ml) and followed by the addition of Na_2_CO_3_ (5 mmol). The solution was refluxed for 2 h. The solid product formed was collected through filtration and then evaporated to dryness. The product was redissolved in MeOH for recrystalliziation as colourless plates. *M. P.*: 145°C.

### Refinement

All N and O bound H atoms were located from the difference map and were refined freely [N–H = 0.857 (19)–0.90 (2) Å and O–H = 0.93 (2) and 0.95 (2) Å]. The remaining H atoms were positioned geometrically and refined using a riding model with *U*_iso_(H) = 1.2 or 1.5*U*_eq_(C) (C–H = 0.9500 and 0.9800 Å). A rotating group model was applied to the methyl groups.

### Crystal data

**Table:**

C_9_H_11_N_3_O_2_	*Z* = 4
*M**_r_* = 193.21	*F*(000) = 408
Triclinic, *P*1	*D*_x_ = 1.394 Mg m^−^^3^
Hall symbol: -P 1	Mo *K*α radiation, λ = 0.71073 Å
*a* = 8.7813 (12) Å	Cell parameters from 3493 reflections
*b* = 9.5432 (13) Å	θ = 2.3–28.5°
*c* = 11.9770 (15) Å	µ = 0.10 mm^−^^1^
α = 80.722 (2)°	*T* = 100 K
β = 78.531 (2)°	Plate, colourless
γ = 70.181 (2)°	0.35 × 0.20 × 0.05 mm
*V* = 920.6 (2) Å^3^	

### Data collection

**Table:**

Bruker APEX DUO CCD diffractometer	4680 independent reflections
Radiation source: fine-focus sealed tube	3815 reflections with *I* > 2σ(*I*)
Graphite monochromator	*R*_int_ = 0.031
φ and ω scans	θ_max_ = 28.6°, θ_min_ = 1.7°
Absorption correction: multi-scan (*SADABS*; Bruker, 2009)	*h* = −11→7
*T*_min_ = 0.965, *T*_max_ = 0.995	*k* = −12→12
12849 measured reflections	*l* = −16→16

### Refinement

**Table:**

Refinement on *F*^2^	Primary atom site location: structure-invariant direct methods
Least-squares matrix: full	Secondary atom site location: difference Fourier map
*R*[*F*^2^ > 2σ(*F*^2^)] = 0.042	Hydrogen site location: inferred from neighbouring sites
*wR*(*F*^2^) = 0.116	H atoms treated by a mixture of independent and constrained refinement
*S* = 1.07	*w* = 1/[σ^2^(*F*_o_^2^) + (0.0538*P*)^2^ + 0.261*P*] where *P* = (*F*_o_^2^ + 2*F*_c_^2^)/3
4680 reflections	(Δ/σ)_max_ < 0.001
287 parameters	Δρ_max_ = 0.34 e Å^−^^3^
0 restraints	Δρ_min_ = −0.26 e Å^−^^3^

### Special details

**Table:**

Experimental. The crystal was placed in the cold stream of an Oxford Cryosystems Cobra open-flow nitrogen cryostat (Cosier & Glazer, 1986) operating at 100 (1) K.
Geometry. All e.s.d.'s (except the e.s.d. in the dihedral angle between two l.s. planes) are estimated using the full covariance matrix. The cell e.s.d.'s are taken into account individually in the estimation of e.s.d.'s in distances, angles and torsion angles; correlations between e.s.d.'s in cell parameters are only used when they are defined by crystal symmetry. An approximate (isotropic) treatment of cell e.s.d.'s is used for estimating e.s.d.'s involving l.s. planes.
Refinement. Refinement of *F*^2^ against ALL reflections. The weighted *R*-factor *wR* and goodness of fit *S* are based on *F*^2^, conventional *R*-factors *R* are based on *F*, with *F* set to zero for negative *F*^2^. The threshold expression of *F*^2^ > σ(*F*^2^) is used only for calculating *R*-factors(gt) *etc*. and is not relevant to the choice of reflections for refinement. *R*-factors based on *F*^2^ are statistically about twice as large as those based on *F*, and *R*- factors based on ALL data will be even larger.

### Fractional atomic coordinates and isotropic or equivalent isotropic displacement parameters (Å^2^)

**Table:**

	*x*	*y*	*z*	*U*_iso_*/*U*_eq_	
O1A	1.06478 (11)	0.25713 (11)	0.46170 (9)	0.0183 (2)	
O2A	0.37005 (11)	0.17841 (12)	0.55131 (8)	0.0200 (2)	
N1A	0.91075 (13)	0.26562 (12)	0.43516 (9)	0.0136 (2)	
N2A	0.91118 (15)	0.50889 (14)	0.36926 (11)	0.0188 (3)	
N3A	0.59696 (13)	0.25477 (13)	0.50343 (9)	0.0132 (2)	
C1A	0.55777 (15)	0.36511 (14)	0.41114 (11)	0.0125 (2)	
C2A	0.40471 (16)	0.41035 (15)	0.37477 (11)	0.0149 (3)	
H2AA	0.3247	0.3646	0.4118	0.018*	
C3A	0.36921 (16)	0.52204 (15)	0.28463 (12)	0.0168 (3)	
H3AA	0.2645	0.5525	0.2613	0.020*	
C4A	0.48421 (17)	0.58940 (15)	0.22839 (11)	0.0167 (3)	
H4AA	0.4596	0.6644	0.1661	0.020*	
C5A	0.63585 (16)	0.54578 (15)	0.26423 (11)	0.0146 (3)	
H5AA	0.7146	0.5924	0.2261	0.018*	
C6A	0.67591 (15)	0.43462 (14)	0.35539 (11)	0.0121 (2)	
C7A	0.83924 (15)	0.39970 (14)	0.39030 (11)	0.0125 (2)	
C8A	0.50778 (15)	0.16825 (14)	0.56596 (11)	0.0134 (3)	
C9A	0.59175 (17)	0.05670 (16)	0.65623 (12)	0.0185 (3)	
H9AA	0.6019	−0.0446	0.6420	0.028*	
H9AB	0.5271	0.0779	0.7318	0.028*	
H9AC	0.7010	0.0641	0.6537	0.028*	
O1B	1.10383 (11)	0.00132 (11)	0.61515 (8)	0.0152 (2)	
O2B	0.71673 (13)	−0.21124 (12)	1.05408 (8)	0.0231 (2)	
N1B	0.97720 (13)	0.02967 (12)	0.71262 (9)	0.0141 (2)	
N2B	1.14247 (14)	0.15404 (13)	0.75828 (11)	0.0161 (2)	
N3B	0.79291 (14)	−0.06646 (13)	0.89487 (10)	0.0159 (2)	
C1B	0.78846 (16)	0.06046 (15)	0.94314 (11)	0.0149 (3)	
C2B	0.68093 (17)	0.10631 (16)	1.04286 (12)	0.0197 (3)	
H2BA	0.6093	0.0513	1.0793	0.024*	
C3B	0.67802 (19)	0.23181 (17)	1.08911 (13)	0.0234 (3)	
H3BA	0.6063	0.2608	1.1580	0.028*	
C4B	0.77935 (18)	0.31493 (17)	1.03513 (13)	0.0223 (3)	
H4BA	0.7768	0.4011	1.0665	0.027*	
C5B	0.88416 (17)	0.27147 (15)	0.93527 (12)	0.0180 (3)	
H5BA	0.9524	0.3295	0.8983	0.022*	
C6B	0.89229 (15)	0.14440 (14)	0.88740 (11)	0.0139 (3)	
C7B	1.01102 (15)	0.10389 (14)	0.78082 (11)	0.0126 (2)	
C8B	0.76306 (17)	−0.19266 (16)	0.95037 (12)	0.0179 (3)	
C9B	0.7947 (2)	−0.31289 (18)	0.87350 (13)	0.0274 (3)	
H9BA	0.7122	−0.3641	0.8981	0.041*	
H9BB	0.9041	−0.3853	0.8780	0.041*	
H9BC	0.7886	−0.2676	0.7944	0.041*	
H3NA	0.696 (2)	0.235 (2)	0.5204 (15)	0.030 (5)*	
H3NB	0.840 (2)	−0.069 (2)	0.8244 (17)	0.028 (5)*	
H1NA	0.998 (2)	0.488 (2)	0.3996 (15)	0.024 (5)*	
H1NB	1.181 (2)	0.165 (2)	0.8176 (18)	0.036 (5)*	
H2NA	0.851 (2)	0.602 (3)	0.3617 (17)	0.038 (5)*	
H2NB	1.218 (2)	0.116 (2)	0.6985 (17)	0.030 (5)*	
H1OA	1.085 (2)	0.168 (2)	0.5101 (17)	0.038 (5)*	
H1OB	1.095 (2)	−0.089 (2)	0.5974 (17)	0.037 (5)*	

### Atomic displacement parameters (Å^2^)

**Table:**

	*U*^11^	*U*^22^	*U*^33^	*U*^12^	*U*^13^	*U*^23^
O1A	0.0131 (5)	0.0187 (5)	0.0250 (5)	−0.0069 (4)	−0.0079 (4)	0.0030 (4)
O2A	0.0145 (5)	0.0259 (5)	0.0208 (5)	−0.0105 (4)	−0.0049 (4)	0.0061 (4)
N1A	0.0104 (5)	0.0155 (5)	0.0161 (5)	−0.0054 (4)	−0.0037 (4)	−0.0003 (4)
N2A	0.0166 (6)	0.0137 (6)	0.0280 (7)	−0.0070 (5)	−0.0077 (5)	0.0023 (5)
N3A	0.0103 (5)	0.0165 (5)	0.0125 (5)	−0.0049 (4)	−0.0020 (4)	0.0010 (4)
C1A	0.0138 (6)	0.0125 (6)	0.0101 (6)	−0.0029 (5)	−0.0008 (5)	−0.0017 (5)
C2A	0.0137 (6)	0.0172 (6)	0.0133 (6)	−0.0044 (5)	−0.0015 (5)	−0.0019 (5)
C3A	0.0141 (6)	0.0188 (7)	0.0164 (7)	−0.0019 (5)	−0.0056 (5)	−0.0023 (5)
C4A	0.0204 (7)	0.0149 (6)	0.0127 (6)	−0.0030 (5)	−0.0047 (5)	0.0011 (5)
C5A	0.0171 (6)	0.0141 (6)	0.0121 (6)	−0.0054 (5)	−0.0004 (5)	−0.0011 (5)
C6A	0.0128 (6)	0.0105 (6)	0.0123 (6)	−0.0020 (5)	−0.0015 (5)	−0.0031 (5)
C7A	0.0135 (6)	0.0137 (6)	0.0104 (6)	−0.0051 (5)	0.0007 (5)	−0.0033 (5)
C8A	0.0125 (6)	0.0145 (6)	0.0123 (6)	−0.0043 (5)	−0.0002 (5)	−0.0013 (5)
C9A	0.0165 (6)	0.0201 (7)	0.0183 (7)	−0.0079 (5)	−0.0039 (5)	0.0057 (5)
O1B	0.0169 (5)	0.0158 (5)	0.0129 (5)	−0.0068 (4)	0.0026 (4)	−0.0036 (4)
O2B	0.0321 (6)	0.0288 (6)	0.0148 (5)	−0.0197 (5)	−0.0053 (4)	0.0044 (4)
N1B	0.0142 (5)	0.0161 (5)	0.0112 (5)	−0.0051 (4)	0.0010 (4)	−0.0019 (4)
N2B	0.0182 (6)	0.0189 (6)	0.0138 (6)	−0.0093 (5)	−0.0020 (5)	−0.0020 (5)
N3B	0.0194 (6)	0.0171 (6)	0.0122 (6)	−0.0087 (5)	−0.0010 (4)	0.0008 (4)
C1B	0.0162 (6)	0.0146 (6)	0.0126 (6)	−0.0029 (5)	−0.0047 (5)	0.0015 (5)
C2B	0.0181 (7)	0.0214 (7)	0.0164 (7)	−0.0045 (5)	−0.0007 (5)	0.0013 (5)
C3B	0.0248 (7)	0.0228 (7)	0.0157 (7)	−0.0004 (6)	0.0016 (6)	−0.0039 (6)
C4B	0.0268 (7)	0.0184 (7)	0.0193 (7)	−0.0031 (6)	−0.0018 (6)	−0.0067 (6)
C5B	0.0199 (7)	0.0160 (6)	0.0175 (7)	−0.0047 (5)	−0.0034 (5)	−0.0012 (5)
C6B	0.0142 (6)	0.0139 (6)	0.0121 (6)	−0.0019 (5)	−0.0043 (5)	0.0001 (5)
C7B	0.0139 (6)	0.0102 (6)	0.0124 (6)	−0.0032 (5)	−0.0028 (5)	0.0020 (4)
C8B	0.0188 (6)	0.0210 (7)	0.0172 (7)	−0.0110 (5)	−0.0055 (5)	0.0030 (5)
C9B	0.0428 (9)	0.0228 (8)	0.0219 (8)	−0.0187 (7)	−0.0041 (7)	−0.0002 (6)

### Geometric parameters (Å, º)

**Table:**

O1A—N1A	1.4232 (13)	O1B—N1B	1.4333 (14)
O1A—H1OA	0.93 (2)	O1B—H1OB	0.95 (2)
O2A—C8A	1.2257 (16)	O2B—C8B	1.2315 (17)
N1A—C7A	1.2992 (17)	N1B—C7B	1.2972 (17)
N2A—C7A	1.3590 (16)	N2B—C7B	1.3577 (16)
N2A—H1NA	0.857 (19)	N2B—H1NB	0.88 (2)
N2A—H2NA	0.87 (2)	N2B—H2NB	0.90 (2)
N3A—C8A	1.3644 (16)	N3B—C8B	1.3593 (17)
N3A—C1A	1.4069 (16)	N3B—C1B	1.4098 (17)
N3A—H3NA	0.885 (19)	N3B—H3NB	0.863 (19)
C1A—C2A	1.4005 (18)	C1B—C2B	1.3962 (19)
C1A—C6A	1.4179 (17)	C1B—C6B	1.4140 (18)
C2A—C3A	1.3915 (18)	C2B—C3B	1.389 (2)
C2A—H2AA	0.9500	C2B—H2BA	0.9500
C3A—C4A	1.3845 (19)	C3B—C4B	1.387 (2)
C3A—H3AA	0.9500	C3B—H3BA	0.9500
C4A—C5A	1.3873 (19)	C4B—C5B	1.383 (2)
C4A—H4AA	0.9500	C4B—H4BA	0.9500
C5A—C6A	1.4027 (17)	C5B—C6B	1.3977 (19)
C5A—H5AA	0.9500	C5B—H5BA	0.9500
C6A—C7A	1.4874 (17)	C6B—C7B	1.4907 (18)
C8A—C9A	1.5031 (18)	C8B—C9B	1.505 (2)
C9A—H9AA	0.9800	C9B—H9BA	0.9800
C9A—H9AB	0.9800	C9B—H9BB	0.9800
C9A—H9AC	0.9800	C9B—H9BC	0.9800
			
N1A—O1A—H1OA	99.4 (12)	N1B—O1B—H1OB	99.3 (12)
C7A—N1A—O1A	109.97 (10)	C7B—N1B—O1B	109.89 (10)
C7A—N2A—H1NA	114.6 (12)	C7B—N2B—H1NB	116.8 (13)
C7A—N2A—H2NA	119.9 (13)	C7B—N2B—H2NB	114.9 (12)
H1NA—N2A—H2NA	117.3 (18)	H1NB—N2B—H2NB	115.9 (17)
C8A—N3A—C1A	129.30 (11)	C8B—N3B—C1B	127.77 (12)
C8A—N3A—H3NA	115.5 (12)	C8B—N3B—H3NB	117.8 (12)
C1A—N3A—H3NA	115.1 (12)	C1B—N3B—H3NB	113.0 (12)
C2A—C1A—N3A	121.95 (11)	C2B—C1B—N3B	121.21 (12)
C2A—C1A—C6A	119.31 (12)	C2B—C1B—C6B	119.76 (12)
N3A—C1A—C6A	118.72 (11)	N3B—C1B—C6B	119.01 (12)
C3A—C2A—C1A	120.31 (12)	C3B—C2B—C1B	120.43 (13)
C3A—C2A—H2AA	119.8	C3B—C2B—H2BA	119.8
C1A—C2A—H2AA	119.8	C1B—C2B—H2BA	119.8
C4A—C3A—C2A	120.99 (12)	C4B—C3B—C2B	120.26 (13)
C4A—C3A—H3AA	119.5	C4B—C3B—H3BA	119.9
C2A—C3A—H3AA	119.5	C2B—C3B—H3BA	119.9
C3A—C4A—C5A	119.06 (12)	C5B—C4B—C3B	119.55 (13)
C3A—C4A—H4AA	120.5	C5B—C4B—H4BA	120.2
C5A—C4A—H4AA	120.5	C3B—C4B—H4BA	120.2
C4A—C5A—C6A	121.70 (12)	C4B—C5B—C6B	121.71 (13)
C4A—C5A—H5AA	119.1	C4B—C5B—H5BA	119.1
C6A—C5A—H5AA	119.1	C6B—C5B—H5BA	119.1
C5A—C6A—C1A	118.61 (11)	C5B—C6B—C1B	118.27 (12)
C5A—C6A—C7A	117.69 (11)	C5B—C6B—C7B	118.22 (12)
C1A—C6A—C7A	123.68 (11)	C1B—C6B—C7B	123.51 (12)
N1A—C7A—N2A	122.40 (12)	N1B—C7B—N2B	124.22 (12)
N1A—C7A—C6A	119.40 (11)	N1B—C7B—C6B	117.67 (11)
N2A—C7A—C6A	118.14 (12)	N2B—C7B—C6B	118.01 (11)
O2A—C8A—N3A	123.82 (12)	O2B—C8B—N3B	124.54 (13)
O2A—C8A—C9A	121.50 (11)	O2B—C8B—C9B	121.64 (13)
N3A—C8A—C9A	114.68 (11)	N3B—C8B—C9B	113.82 (12)
C8A—C9A—H9AA	109.5	C8B—C9B—H9BA	109.5
C8A—C9A—H9AB	109.5	C8B—C9B—H9BB	109.5
H9AA—C9A—H9AB	109.5	H9BA—C9B—H9BB	109.5
C8A—C9A—H9AC	109.5	C8B—C9B—H9BC	109.5
H9AA—C9A—H9AC	109.5	H9BA—C9B—H9BC	109.5
H9AB—C9A—H9AC	109.5	H9BB—C9B—H9BC	109.5
			
C8A—N3A—C1A—C2A	5.0 (2)	C8B—N3B—C1B—C2B	−31.7 (2)
C8A—N3A—C1A—C6A	−176.47 (12)	C8B—N3B—C1B—C6B	149.88 (14)
N3A—C1A—C2A—C3A	178.69 (12)	N3B—C1B—C2B—C3B	−179.89 (13)
C6A—C1A—C2A—C3A	0.20 (19)	C6B—C1B—C2B—C3B	−1.5 (2)
C1A—C2A—C3A—C4A	0.7 (2)	C1B—C2B—C3B—C4B	1.5 (2)
C2A—C3A—C4A—C5A	−1.0 (2)	C2B—C3B—C4B—C5B	−0.4 (2)
C3A—C4A—C5A—C6A	0.4 (2)	C3B—C4B—C5B—C6B	−0.7 (2)
C4A—C5A—C6A—C1A	0.45 (19)	C4B—C5B—C6B—C1B	0.7 (2)
C4A—C5A—C6A—C7A	−177.74 (12)	C4B—C5B—C6B—C7B	−178.83 (12)
C2A—C1A—C6A—C5A	−0.76 (18)	C2B—C1B—C6B—C5B	0.42 (19)
N3A—C1A—C6A—C5A	−179.30 (12)	N3B—C1B—C6B—C5B	178.85 (12)
C2A—C1A—C6A—C7A	177.32 (12)	C2B—C1B—C6B—C7B	179.89 (12)
N3A—C1A—C6A—C7A	−1.22 (18)	N3B—C1B—C6B—C7B	−1.69 (19)
O1A—N1A—C7A—N2A	2.73 (17)	O1B—N1B—C7B—N2B	3.63 (17)
O1A—N1A—C7A—C6A	179.87 (10)	O1B—N1B—C7B—C6B	179.80 (10)
C5A—C6A—C7A—N1A	−150.83 (12)	C5B—C6B—C7B—N1B	−152.96 (12)
C1A—C6A—C7A—N1A	31.08 (18)	C1B—C6B—C7B—N1B	27.57 (18)
C5A—C6A—C7A—N2A	26.43 (17)	C5B—C6B—C7B—N2B	23.45 (18)
C1A—C6A—C7A—N2A	−151.66 (13)	C1B—C6B—C7B—N2B	−156.02 (12)
C1A—N3A—C8A—O2A	−2.2 (2)	C1B—N3B—C8B—O2B	4.6 (2)
C1A—N3A—C8A—C9A	177.71 (13)	C1B—N3B—C8B—C9B	−174.30 (13)

### Hydrogen-bond geometry (Å, º)

Cg1 and Cg2 are the centroids of the C1A–C6A and C1B–C6B benzene rings, respectively.

**Table:**

*D*—H···*A*	*D*—H	H···*A*	*D*···*A*	*D*—H···*A*
N3*A*—H3*NA*···N1*A*	0.883 (19)	2.048 (19)	2.7463 (18)	135.2 (15)
N3*B*—H3*NB*···N1*B*	0.86 (2)	1.963 (19)	2.6798 (17)	139.7 (17)
N2*B*—H1*NB*···O2*B*^i^	0.88 (2)	2.10 (2)	2.9725 (17)	173.0 (17)
N2*A*—H2*NA*···O2*A*^ii^	0.87 (3)	2.53 (2)	3.3004 (17)	149.0 (17)
N2*A*—H2*NA*···N2*B*^iii^	0.87 (3)	2.54 (2)	3.2522 (18)	139.6 (17)
N2*B*—H2*NB*···O2*A*^iv^	0.903 (19)	2.12 (2)	2.8900 (17)	142.1 (16)
O1*A*—H1*OA*···O1*B*	0.933 (19)	1.844 (19)	2.7733 (15)	173.9 (18)
O1*B*—H1*OB*···N1*A*^v^	0.951 (18)	1.809 (18)	2.7597 (15)	177.0 (17)
C2*A*—H2*AA*···O2*A*	0.95	2.22	2.8556 (17)	123
C5*A*—H5*AA*···O2*B*^vi^	0.95	2.55	3.2773 (17)	133
C9*A*—H9*AC*···N1*B*	0.98	2.56	3.499 (2)	160
C4*A*—H4*AA*···*Cg*2^ii^	0.95	2.95	3.7524 (16)	143
C3*B*—H3*BA*···*Cg*1^vii^	0.95	2.88	3.6645 (17)	141

Symmetry codes: (i) −*x*+2, −*y*, −*z*+2; (ii) −*x*+1, −*y*+1, −*z*+1; (iii) −*x*+2, −*y*+1, −*z*+1; (iv) *x*+1, *y*, *z*; (v) −*x*+2, −*y*, −*z*+1; (vi) *x*, *y*+1, *z*−1; (vii) *x*, *y*, *z*+1.
